# Tissue-engineered bone construct promotes early osseointegration of implants with low primary stability in oversized osteotomy

**DOI:** 10.1186/s12903-023-03834-x

**Published:** 2024-01-10

**Authors:** Lianyi Xu, Reinhilde Jacobs, Yingguang Cao, Xiaojuan Sun, Xu Qin

**Affiliations:** 1 Department of Stomatology, Tongji Hospital, Tongji Medical College, Huazhong University of Science and Technology, Wuhan, 430030 Hubei China; 2 School of Stomatology, Tongji Medical College, Huazhong University of Science and Technology, Wuhan, 430030 Hubei China; 3 Hubei Province Key Laboratory of Oral and Maxillofacial Development and Regeneration, Wuhan, 430022 Hubei China; 4 Department of Imaging and Pathology, OMFS-IMPATH, KU Leuven, Kapucijnenvoer 7, Leuven, 3000 Belgium; 5 Department of Dental Medicine, Karolinska Institutet, Stockholm, SE-171 77 Sweden; 6 Department of Oral and Maxillofacial Surgery, General Hospital, Ningxia Medical University, 804 Shengli Street, Yinchuan, 750004 China

**Keywords:** Dental implant, Primary stability, Osseointegration, Animal study, Tissue engineered bone graft

## Abstract

**Objectives:**

To evaluate the histological parameters and bone mechanical properties around implants with low primary stability (PS) in grafted bone substitutes within an oversized osteotomy.

**Materials and methods:**

An oversized osteotomy penetrating the double cortical bone layers was made on both femora of 24 New Zealand white rabbits. Bilaterally in the femur of all animals, 48 implants were installed, subdivided into four groups, corresponding to four prepared tissue-engineering bone complexes (TEBCs), which were placed between the implant surface and native bone wall: A: tricalcium phosphate β (TCP-β); B: autologous adipose derived-stem cells with TCP-β (ASCs/TCP-β); C: ASCs transfected with the enhanced-GFP gene with TCP-β (EGFP-ASCs/TCP-β); D: ASCs transfected with the BMP-2 gene with TCP-β (BMP2-ASCs/TCP-β). Trichrome fluorescent labeling was conducted. Animals were sacrificed after eight weeks. The trichromatic fluorescent labeling (%TFL), area of new bone (%NB), residual material (%RM), bone-implant contact (%BIC), and the removal torque force (RTF, N/cm) were assessed.

**Results:**

ASCs were successfully isolated from adipose tissue, and the primary ASCs were induced into osteogenic, chondrogenic, and adipogenic differentiation. The BMP-2 overexpression of ASCs sustained for ten days and greatly enhanced the expression of osteopontin (OPN). At eight weeks post-implantation, increased %NB and RTF were found in all groups. The most significant value of %TFL, %BIC and lowest %RM was detected in group D.

**Conclusion:**

The low PS implants osseointegrate with considerable new bone in grafted TEBCs within an oversized osteotomy. Applying BMP-2 overexpressing ASCs-based TEBC promoted earlier osseointegration and more solid bone mechanical properties on low PS implants. Bone graft offers a wedging effect for the implant with low PS at placement and promotes osteogenesis on their surface in the healing period.

## Introduction

Primary stability (PS) has long been considered an essential requirement for the osseointegration of dental implants and their long-term success [1]. Defined as the biometric stability immediately following the insertion, PS reflects the mechanical engagement of an implant with the surrounding bone [2]. In the classic implantation paradigm of Branemark, implant immobility during the first three to six months of healing is a prerequisite for osseointegration. The reason is that implant micromovements caused by functional forces during wound healing may induce fibrous tissue formation rather than osseointegration [3]. There have been extensive studies on the influence elements of PS. These studies detected the relationship of PS with bone density and quality, implant profile and thread design, treatment plan adjustment, immediate loading judgment, and surgical protocol improvement [4–6]. A well-known implant stability curve was summarized in 2005 to correlate reducing PS with increasing secondary stability (SS) to reflect the dynamic change of total stability (TS) [7]. In general, most studies were based on a consensus that various strategies need to be developed to secure a considerable PS, which plays a vital role in ensuring the early success of osseointegration. On the other hand, the influence of low PS on the early success rate of dental implantation has become disputable [8–10].

Low PS is sometimes unavoidable in clinics because of anatomical, pathological, and iatrogenic issues. It tends to occur in patients with systematic diseases leading to bone metabolism problems, immediate implantation situations in the oversized implant bed caused by an improper drilling protocol, prolonged inflammation in alveolar bone and fresh extraction socket, or delayed implantation in challenging bone conditions, such as posterior maxillary site [11, 12]. To tackle low PS, the bone substitute is routinely recommended in the circumstances described above to create a wedging effect of the implant within the peri-implant defect [13, 14]. However, autologous bone has limited sources, artificial grafts still have problems like insufficient osteoinductive capability [15].

Tissue Engineering (TE) tunes three elements, including scaffold, growth factors, and seed cells to compose a viable integrity to repair, improve, and replace the damaged/missing parts of the organism [16]. Over the years, the optimization of tissue engineering bone complex (TEBC) has been greatly propelled to handle the craniofacial bone defect via stem cell selection and bioactive factor delivery [17–19]. Adipose-deprived mesenchymal stem cells (ASCs) secrete various growth factors to promote wound healing and to regenerate various tissues. They can be easily harvested from subcutaneous adipose tissue in clinical practice under local anesthesia [20]. In the past decades, plenty of preclinic and clinic studies have proved the safety and efficiency of ASCs loaded TEBCs in craniofacial bone regeneration [21–23]. Bone morphogenic protein 2 (BMP-2) plays a central role in bone-tissue engineering because of its potent osteoinductive ability [24]. Sandor et al. applied TEBC of ASCs, β-TCP and BMP-2 to treat a large anterior mandibular defect through early dental implant osseointegration [25]. Though BMP-2 is the strongest inducer of osteogenesis, its short half-life and quick release in vivo brought up problems like supra-physiological dosage and large medical expense [19]. The application of gene therapy might be of special advantage for inducing robust in situ expression of BMP-2 protein to promote precise bone regeneration [15].

The direct measurement of PS is still challenging due to the difficulty of evaluating the mechanical force change on the interface between the implant body and the native bone [2]. Several methods, including cutting torque resistance analysis (CRA), insertional torque (IT), periotest, and resonance frequency analysis (RFA), have been developed to quantify implant stability. And these methods, each reflecting a particular characteristic of PS, are independent and incomparable ways to represent implant-to-bone contact conditions as soon as an implant is inserted [5, 26–28].

In this study, we harvested autologous ASCs to construct a BMP-2 overexpressing TEBC. A damaged socket was made on the rabbit femur by making a penetrating osteotomy. The histological parameters and bone mechanical properties around implants with low PS in grafted bone substitutes within the oversized osteotomy was evaluated.

## Materials & methods

### Animals

All experiments involving animals were carried out in accordance with the Guidelines of the Animal Ethics Committee of Tongji Hospital, Huazhong University of Science and Technology Institutional Review Board (IRB ID: 20,171,019). The study complied with the ARRIVE guidelines for preclinical animal studies [29]. Twenty-four healthy New Zealand white rabbits of 3 months old in healthy condition, each weighing 2.5-3 kg, male, were obtained from the animal care center of Tongji Medical College. All animals were kept in separate cages under identical standard conditions. General anesthesia of ketamine (10 mg/kg) (Shanghai Huatai Chemical Co. Ltd., Shanghai, China) and xylazine (3 mg/kg) (Shanghai Demo Medical Tech Co. Ltd., Shanghai, China) was taken before adipose tissue isolation and implant operations. Adequate measures were implemented to minimize the pain or discomfort of animals during all surgical procedures [30].

### ASCs isolation and the multi-lineage differentiation inducement

Subcutaneous adipose tissue was harvested from the inguinal fat pad of all rabbits 3 weeks before implantation surgery. After general anesthesia, the animal was fixed in a supine position. The surgical area was then shaved and disinfected by iodophor cotton ball (Anda Health Industry Co. Ltd, Yangzhou, China). A 3 cm cut was made on the skin at a distance of 1.5 cm parallel to the groin in the lower abdomen. Bluntly separated the fascia and obtained 2 cm ×3 cm subcutaneous adipose tissue. The fresh tissue was placed in a sterilized stainless bowl for thoroughly washing with PBS. Then, the well-minced adipose tissue was digested by 0.1% collagenase at 37 ˚C for one hour (type II-S; Sigma-Aldrich, St. Louis, MO, USA). Digestion activity was terminated by supplemented Dulbecco’s modified Eagle’s medium (DMEM) (Gibco Biocult Co., Paisley, Strathclyde, U.K.) with 10% fetal bovine serum (Hyclone, Logan, UT, USA), 100U/mL penicillin, and 100 mg/mL streptomycin) (Shanghai Xianfeng Pharmaceutical Factory, Shanghai, China). The obtained chyle-like tissue was centrifuged at 1000 rpm for 10 min to discard the supernatant tissue. Washed twice with PBS, the cell suspension was seeded in a 100-mm culture plate at a density of 1 × 10^5^ cells/ml and then cultured in supplemented DMEM at 37 ℃ in a humidified 5% CO_2_ incubator. Non-adherent cells were removed three days later by medium change, and the adherent cells were propagated twice weekly [31]. Cells in passages 2–5 were used for the following study [20].

Osteogenic, adipogenic, and chondrogenic lineage differentiation tests were conducted to identify the multi-differentiation potentials of ASCs. For osteogenic differentiation, primary cells were seeded to a 24-well plate at a density of 8.0 × 10 ^3^/cm^2^ in supplemented DMEM. When the cells fusion reached 100%, the culture medium was replaced to osteogenic medium (DMEM, 10% FBS, 1% penicillin/streptomycin, 50 µg/mL L-ascorbic acid, 10 mM glycerophosphate and 100 nM dexamethasone) (Sigma-Aldrich, USA). Changed the osteogenic medium every three days. Alizarin red S staining (Sigma-Aldrich, USA) was conducted for calcified nodules detection. For adipogenic differentiation, primary cells were seeded at a density of 8.0 × 10 ^3^/cm^2^ on a 24-well culture plate. When the fusion rate reached 60%, replace the supplemented DMEM with adipogenic medium (supplemented DMEM with 0.5 mM isobutylmethylxanthine, 0.5 mM hydrocortisone and 60 mM indomethacin) (Cyagen Biosciences Inc., Guangzhou, Guangdong, China). The medium was changed once every three days. On the 28th day, the cells were stained with 2% oil red O (Cyagen, China) [32]. For chondrogenic culture, a total of 2.0 × 10^5^ cells were collected into a 15 mL centrifuge tube and centrifuged at 1500 r/min for 10 min to precipitate at the bottom of the culture tube. Chondrogenic medium containing 10% FBS, 1% penicillin/streptomycin, 1% ITS, 0.1 mM L-ascorbate-2-phosphate, 0.4 mM proline, 100 nM dexamethasone and 10 ng/ml transforming growth factor-β3 (TGF-β3) (Cyagen, China) was added and placed in a 37 ℃ humidified 5% CO_2_ incubator. The medium was half changed every three days. On the 21st day of cultivation, the cell clusters were fixed with 4% paraformaldehyde and identified by Alcian blue staining (Cyagen, China) after sectioning [33]. Cells living on the culture plate substrate were released with trysin/EDTA and centrifuged at 1000 rpm for 5 min. After PBS wash, 1.0 × 10^6^ primary cells were incubated in the dark with cell-surface antigens at room temperature for the detection of CD34, CD44, CD45, CD105, and CD11b (Invitrogen, Carlsbad, California, USA) by flow cytometry, as described previously [34].

### Ad-BMP-2 gene transduction

Primary cells were seeded to a 6-well plate at a density of 5 × 10^4^ cells/ml to achieve a fusion rate of 80% the next day. The recombinant replication defective adenovirus with BMP-2 (AdBMP-2) was adopted for the gene transduction under a multiplicity of infection (M.O.I.) of 50 pfu/cell based on our previous study. The recombinant replication-defective adenovirus with an enhanced green fluorescent protein (EGFP) was transfected under the identical method as a cell model control of gene transfer efficiency [20]. The infected ASCs were maintained in supplemented DMEM in a humidified 5% CO_2_ incubator.

### In vitro expression of BMP-2

In vitro secretion of BMP-2 by BMP-2 gene-enhanced ASCs was assessed by the enzyme-linked immunosorbent assay (ELISA). BMP-2/ASCs were plated in a 24-well plate at a density of 5 × 10^4^ cells/well before BMP-2 transduction. The transduction was performed according to our previous protocols [20]. ASCs prepared at the same time for the control. The culture medium was collected at 1, 4, 7, 10, 14, 21, and 28 days after transduction. The collected media froze at -80 ℃ before the final analysis. A commercial ELISA kit (Quantikine BMP-2 microplate, R&D systems, Minneapolis, MA, USA) coated with the mouse monoclonal antibody against BMP-2 was adopted for BMP-2 concentration measurement. All experiments were performed in triplicate [35, 36].

### Osteogenic differentiation

Immunofluorescence staining of OPN was applied 14 days after transfection. BMP-2 overexpressing ASCs were seeded on sterile glass coverslips loaded on the bottom of a 6-well plate at a density of 1 × 10^2^ cells/well. After osteogenic medium culture for 14 days, the cells were washed twice with cold PBS and fixed in 4% paraformaldehyde for 15 min at 4 ˚C. Cells were then treated with 0.3% Triton-X100 (Sigma-Aldrich, USA) for 30 min and blocked in 3% BSA (Sigma-Aldrich, USA) at room temperature for 30 min. Specific primary antibodies targeting rabbit OPN (Abcam, Cambridge, MA, USA) were added to the fixed cells at a dilution of 1:100 and incubated at 4 ˚C overnight. A fluorescent labeling phycoerythrin (PE) goat anti-mouse secondary antibody (Abcam, USA) was incubated with the cells at a dilution of 1:500 in blocking buffer at 37 ˚C in the dark for 1 h. Nuclei were stained with DAPI (Invitrogen, USA) in the dark for another 5 min. The specimens were examined under a confocal laser scanning microscope (CLSM; Leica TCS Sp2 AOBS, Germany).

### Tissue-engineered bone constructs preparation (TEBCs)

Untreated and transfected ASCs were digested by trypsin/EDTA (Sigma–Aldrich, USA) three days after gene transduction. The cell suspensions were concentrated to a density of 2 × 10^7^ cells/ml in DMEM. The proper volume of the cell suspension was slowly pipetted onto β-TCP without spilling until a final saturation [37]. The complexes were then incubated for an additional 4 h for cell attachment. Four groups of tissue-engineered bone grafts were prepared as: A: β-TCP (n = 12), B: ASCs/β-TCP (n = 12), C: EGFP/ASCs/β-TCP (n = 12), and D: BMP-2/ASCs/β-TCP (n = 12).

### Implant surgery

**Fig. 1:**
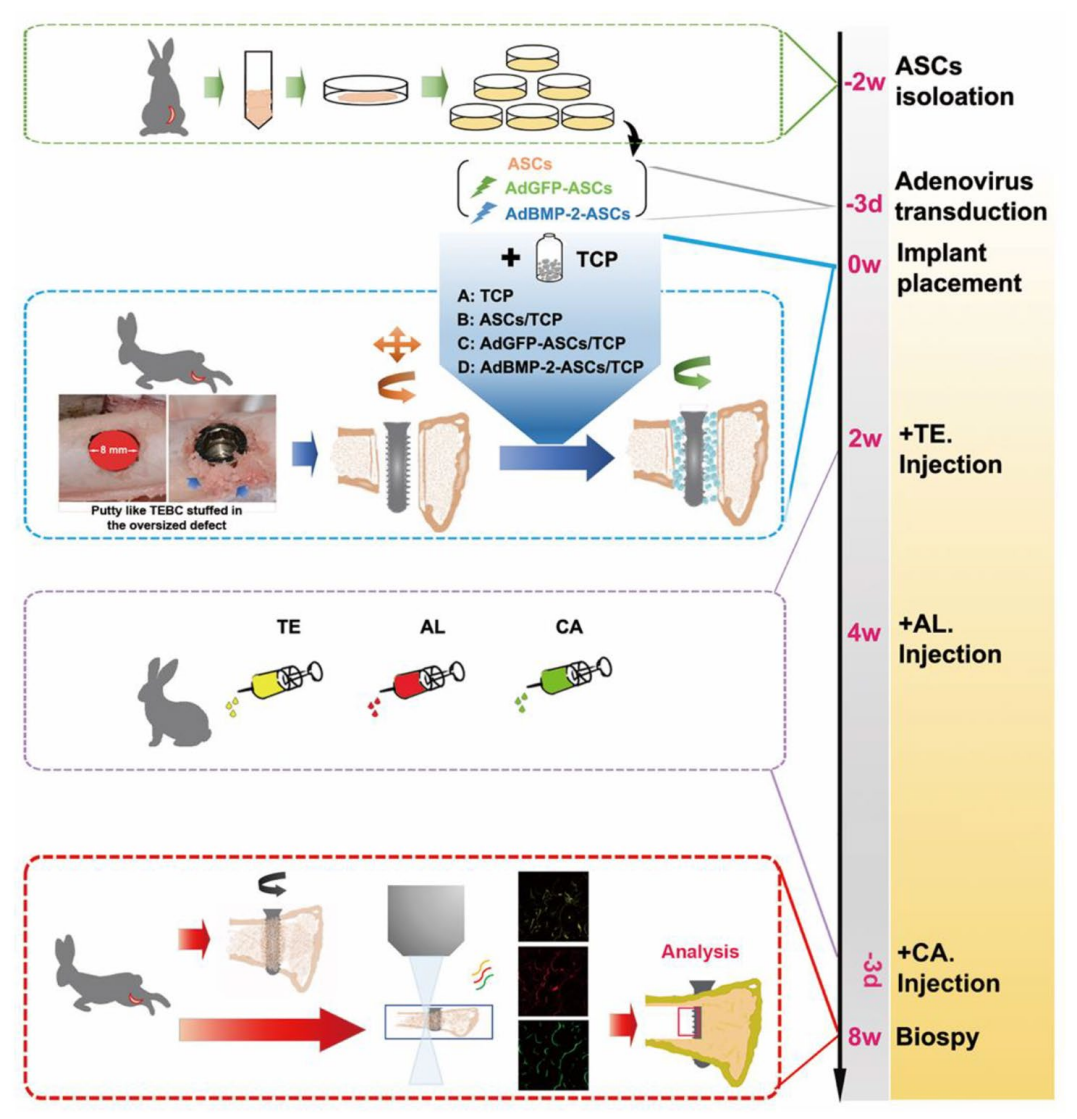
Schematic illustration of the study. An oversized implant site osteotomy (8 mm diameter) was made in the rabbit femur. An implant diameter of 3.8 mm was placed in the prepared site with low PS. (ASCs: adipose-derived mesenchymal stem cells, TE: tetracycline, AL: alizarin red s, CA: calcein)

All rabbits were anesthetized with 0.5 mg/kg sodium pentobarbital intravenously. Then 0.5 ml of 1% lidocaine with epinephrine (1:100,000) was injected subcutaneously for local anesthesia. After anesthetization, a 6 cm long incision was made along the distal end of the femur with a NO. 15 blade. The skin, the subcutaneous tissue, and the muscle were drawn back to expose the bone surface. An implant bed was prepared stepwise through the double cortex and perpendicular to the femur shaft without entering the distal femoral condyle (Fig. 1). After the last drill, a slow-speed burr was used to enlarge the double cortical defects to the diameter of 8 mm [38]. Once made the implant bed, the prepared TEBC was gradually stuffed into the space. The implant (10 mm height, 3.8 mm diameter of the implant body, China Dental Implantology Center, Sichuan, China) was then inserted into the TEBC without touching the rest cortical bone border. The prepared TEBC was pushed into the space between the implant surface and the residual bone wall to keep the implant’s stability within the oversized site. The implant insertion depth was controlled, which sets the implant neck well below the upper edge of the cortical bone. The TEBCs were placed in a randomized order. The surgeons were blinded regarding TEBC type. The implant insertion torque was measured at the time of placement employing the Surgic XT Plus^™^ (NSK, Kanuma, Japan) device [3]. The soft tissue was carefully repositioned and sutured in different layers for primary wound closure using an absorbable suture (Poligalactina 910 – Vycril 4.0, Ethicon, Johnson Prod., NJ, USA), leaving the implants submerged. Monofilament suture (Nylon 5.0, Ethicon, Johnson, NJ, USA) was used for interrupted skin suturing [39].

### Trichromatic fluorescent labeling

The trichromatic sequential fluorescent labeling was implemented to reflect the active process of new bone formation [19]. Two, four, and eight weeks after the operation, the animals were intraperitoneally administered 25 mg/kg hydrochloride tetracycline (TE), 30 mg/kg alizarin red s (AL), and 20 mg/kg calcein (CA, Sigma-Aldrich, USA), respectively.

### Animal sacrifice and perfusion

Animals were sacrificed at eight weeks post-operation (3 days after the injection of CA) and perfused with 10% buffered formalin. The femora were cut into single blocks with one implant before storage in a 4% neutral formaldehyde solution at 4 ℃ [30, 37, 40].

### Histological and histomorphological analysis

Calibration between examiners (LX, XQ) was performed prior to the histological analysis. The examiners were blinded regarding TEBC type and healing time. Half samples were gradually dehydrated and embedded in methyl methacrylate-based resin (Technovit 7200 VLC, Kulzer, Friedrichsdorf, Germany). The implant blocks were cut along the mesiodistal direction (ExaktA, Parenteau, Norderstedt, Germany). Three central sections were prepared of one implant and subsequently polished to 200 μm for trichrome fluorescent labeling observation. Five sites adjacent to the implant surface were chosen.

The areas of single and total trichrome fluorescent labeling were evaluated. Trichrome fluorescent labeling was observed under CLSM on all sections. Four sites adjacent to the implant surface were chosen. The excitation/emission wavelengths were 405/580 nm (TE), 543/617 nm (AL), and 488/517 nm (CA). The bone formation indices were evaluated on a picture-analysis system (Image Pro 5.0, Media Cybernetic, MD, USA), and the areas of trichrome fluorescent labeling (TFL%) were measured by calculating the mean value of the images taken around the implant surface on the sections.

**Fig. 2:**
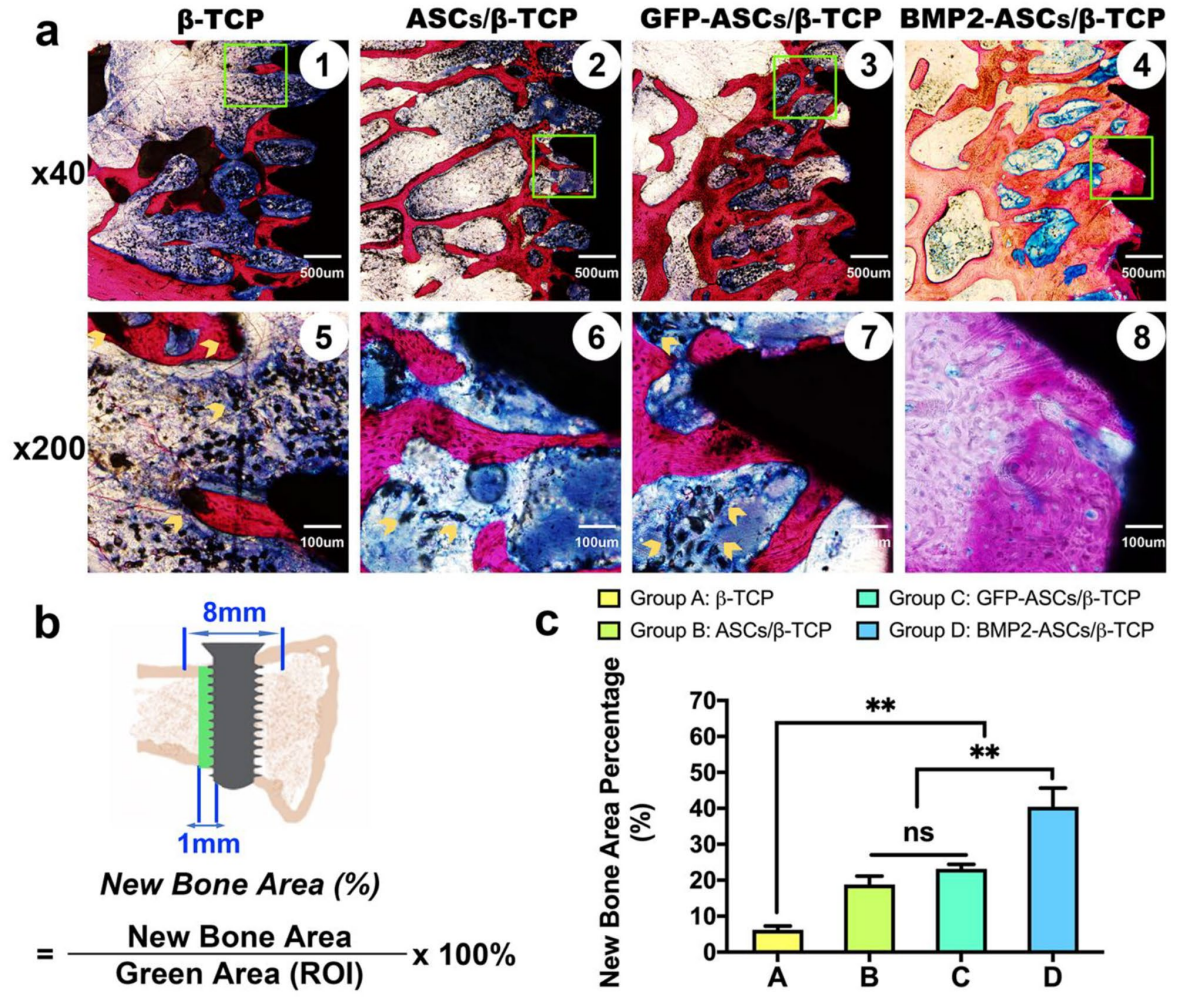
New bone formation. **a**: Histological observation of Van Gieson’s picro-fuchsin staining (VG staining) of four groups after eight weeks of healing. **b**: Region of interest (ROI) for the new bone area. **c**: Histomorphological result of %NB. (**, *p* < 0.01, ns, no significant difference) (yellow arrows, the residual grafted bone substitutes)

**Fig. 3:**
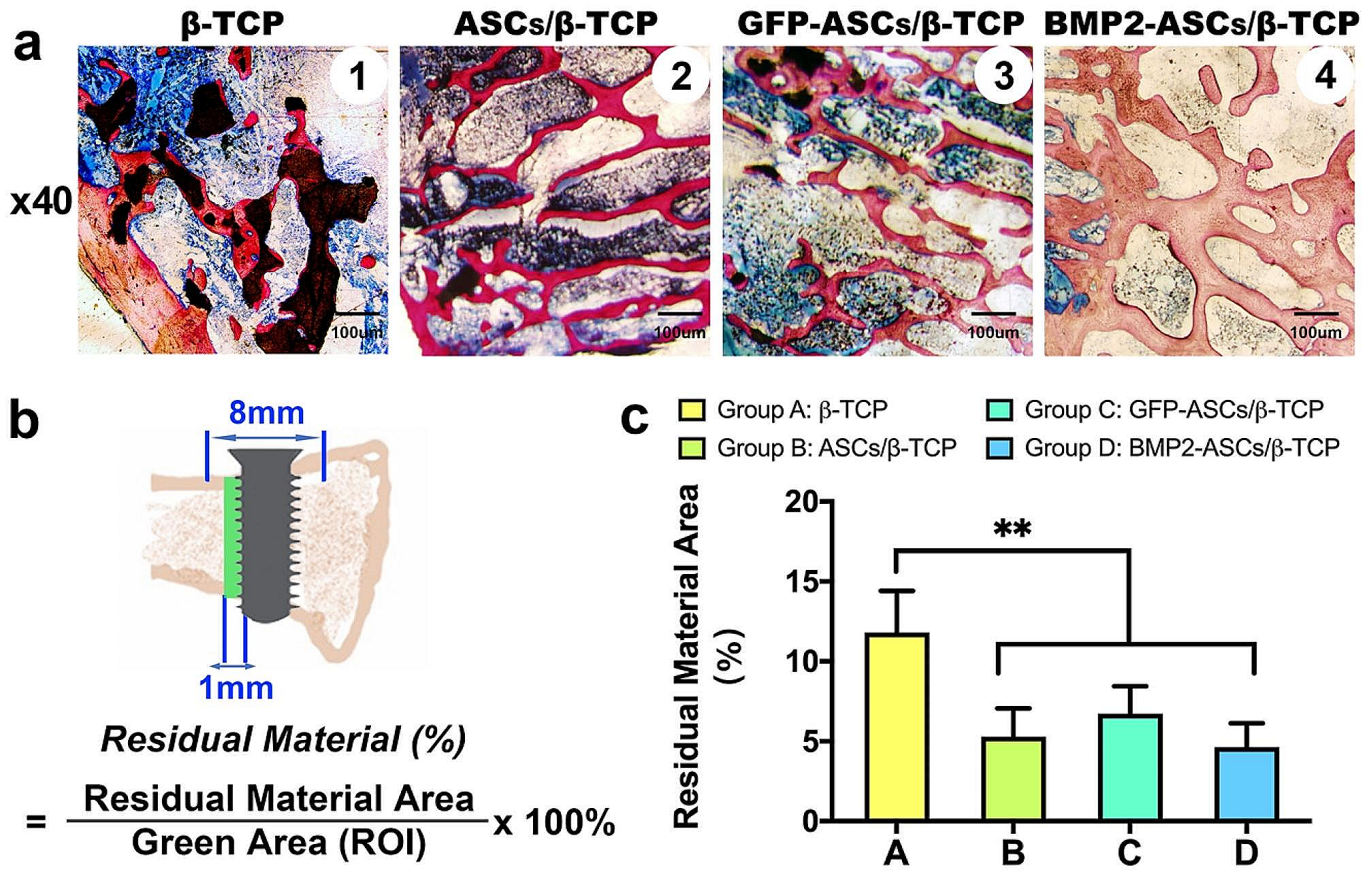
Residual Material. **a**: Histological observation of the residual material of four groups after eight weeks of healing. **b**: ROI for the residual material. **c**: Histomorphological result of %RM. (**, *p* < 0.01)

These sections were then polished to 40 μm. Van Gieson’s picro-fuchsin (VG) staining was performed on these finely polished sections. Histomorphometric measurements were performed blinded and carried out using Image Pro 5.0. The following parameters were measured: new bone area (NB, %): the percentage of new bone area to the region of interest (ROI) on the central section crossing the mesiodistal direction of the implant (Fig. 2b). The ROI area was the one cm width zone extending from the implant surface to concentration on the new bone deposition on the implant. The area encompassed the peri-implant tissue in both the cortical and medullar regions at the distal side of the implant. Residual material (RM, %): the percentage of residual material area to the ROI (Fig. 3b). Bone contact with implant (BIC, %): the portion of new bone contact to the implant surface: summation of the lengths of contact between the implant and the host bone/implant length corresponding to the width between the double cortical bone. Sections were imaged with an Olympus BX-51 microscope, and cross-sections were analyzed using the Image J software package.

### Removal torque tests

The removal torque test was applied to assess the SS of the integrated implant in the bone [41]. The removal torque value (RTV, N/cm) reflects the interfacial shear strength. Static torque was applied to the implant by a machine-run gradual increase at a linear rate of 9.5 Ncm/s. A rotational unscrewing force was applied, and the strength was determined as the peak force applied to loosen the implant from the bone as measured with a digital torque meter (MGT20Z, Mark-10 Corp., New York, NY).

### Statistical analysis

The data are presented as mean ± standard deviation (SD) from at least three independent experiments. Statistically significant differences (*p* < 0.05) between the different groups were measured using a one-way analysis of variance with Tukey post hoc analysis when indicated. All statistical analysis was completed by a SAS 6.12 statistical software package (SAS, Cary, NC, USA).

## Results

No signs of infection were displayed after the implant insertion surgery. All 48 implants were clinically stable at the time of animal euthanasia. The gross observation showed successful osseointegration and significant interfacial remodeling for four groups. Histological parameters and bone mechanic properties were quantified to gain insight into the healing process around these implants in the grafted osteotomy.

### Rabbit ASCs culture and multipotency characterization

**Fig. 4:**
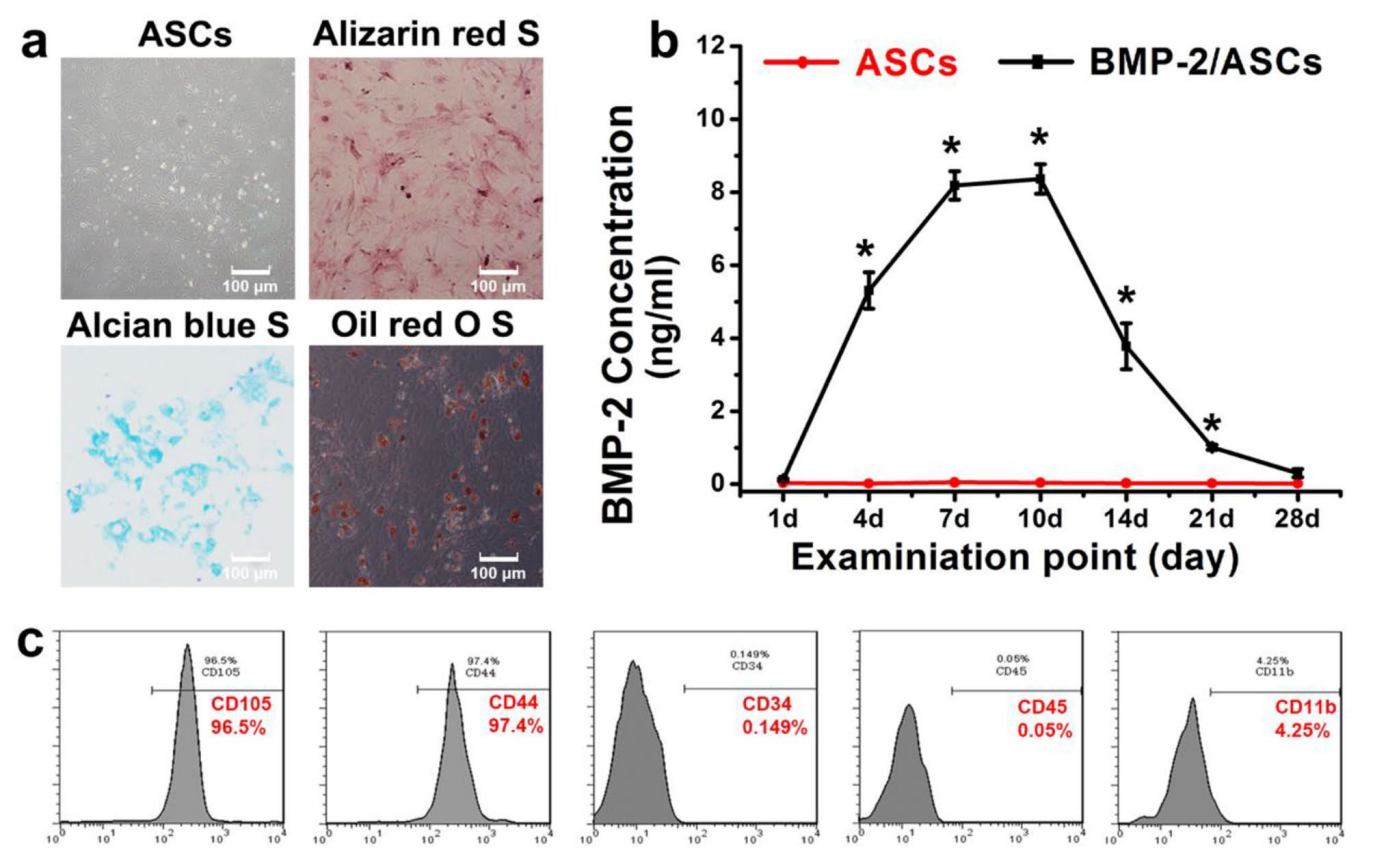
Primary culture and multi-lineage differentiation tests of the isolated adipose-derived stem cells (ASCs). **a**: In vitro culturing ASCs at passage three; calcified nodules detected by Alizarin red S staining after four weeks inducement; lipid droplets within cells detected by oil red O staining after 28 days inducement; cartilage proteoglycan detected by Alcian blue staining after three weeks inducement. **b**: The amount of BMP-2 secreted by ASCs at sequent time points was determined by ELISA assay. Non-transduced ASCs were used as the control. The value at each time point represents the concentration of BMP-2 in the harvested media at the time point when the media was replaced (*, *p* < 0.05). **c**: The figure shows flow cytometry data for ASCs. The harvested cells expressed surface markers associated with stem cell function, such as CD105 and CD44; barely expressed CD34, CD45, or CD11b

ASCs were successfully isolated from adipose tissue, and primary ASCs presented a fibroblast-like spindle shape. The multiple lineage differentiation tests demonstrated that the isolated ASCs could differentiate into osteoblasts, adipocytes, and chondrocytes which stained positive for mineral nodules with Alizarin red S, for lipid droplets with Oil Red O, and cartilage proteoglycan with Alcian blue, respectively (Fig. 4a). The flow cytometry data of ASCs presented the expressed surface markers associated with stem cell function, such as CD105 and CD44. Meanwhile, these cells barely expressed CD34, CD45, or CD11b (Fig. 4c). The primary ASCs were amplified for the preparation of TEBGs of group B, C, and D.

### BMP-2 secretion and osteogenic differentiation

**Fig. 5:**
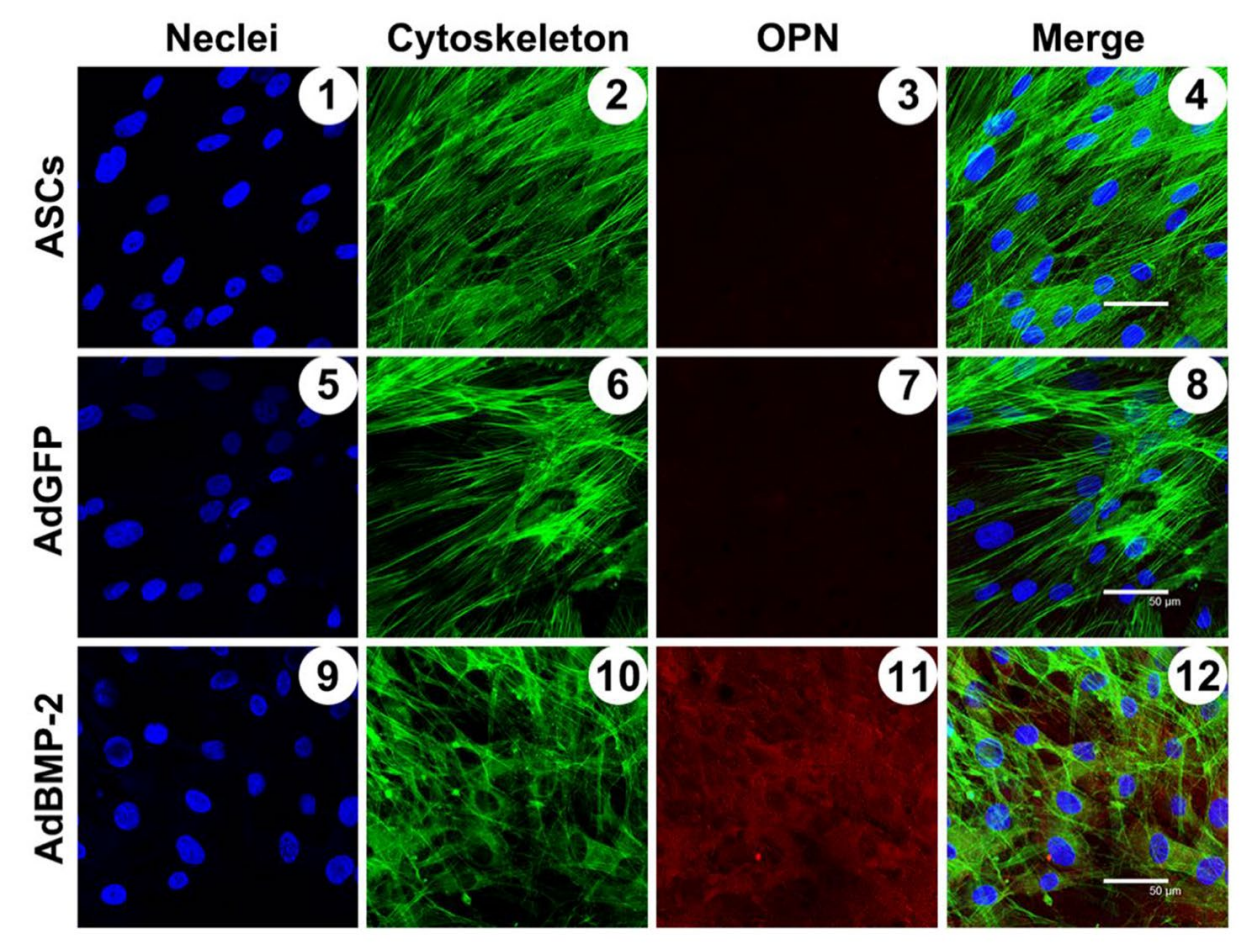
Immunofluorescence staining of OPN. Fourteen days after transfection, the OPN expression was detected by immunofluorescence to indicate the osteogenic differentiation of ASCs (**1–4**), EGFP/ASCs (**5–8**), and BMP-2/ASCs (**9 – l2**). The nuclei are stained blue by DAPI; the cytoskeleton is green as stained by phalloidin; OPN secretions are listed in the third column. The highest secretion is in the BMP-2/ASCs, while limited expression in the ASCs and EGFP/ASCs; The fourth column shows the merged images of the nuclei, skeleton, and target protein, OPN

As shown in Fig. 4b, BMP-2/ASCs consistently secreted BMP-2 during the entire period of analysis. The maximum concentration appeared at 7d post-transduction and was sustained for about three days (day 10). Then, a sharp decrease in BMP-2 secreting was noticed, and the ASCs produced no detectable BMP-2. Osteopontin (OPN), synthesized by mature osteoblasts, plays a vital role in hard tissue formation. Immunofluorescence detected that 14 days after transfection, OPN secretion was significantly increased in BMP2-ASCs (Fig. 5). There was a limited expression of the ASCs group and the EGFP-ASCs. These results indicated that the osteogenic differentiation capability of AdBMP-2-ASCs has been promoted at an early stage (within 14 days) compared to control ASCs.

### Trichrome fluorescent labeling results

**Fig. 6:**
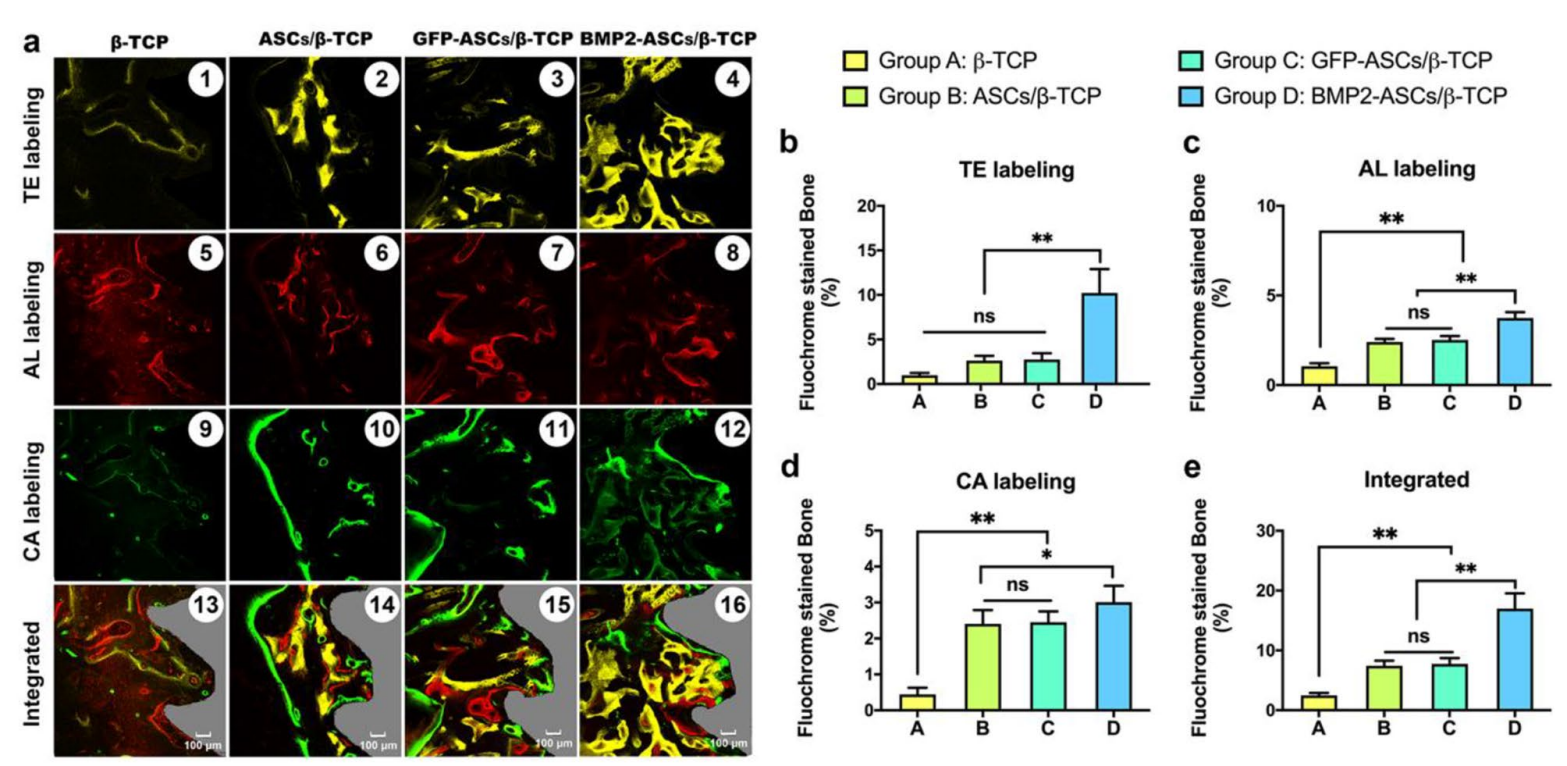
Trichromatic fluorescent labeling assay. **a**: Trichromatic fluorescent labeling of the four groups 2-, 4-, and 8-weeks post-implantation. Newly formed bones infiltrated into and deposited on the implant’s surface were observed. The most potent calcified mineralization shows in group D. **b**: TE labeling result; **c**: AL labeling result; **d**: CA labeling result; **E**: Integrated labeling result. (*, *p* < 0.05, **, *p* < 0.01, ns, no significant difference)

Sequential fluorescent labeling analysis could trace the calcium deposition activity during bone healing. For the area of labeled single chrome (Fig. 6a. TE%, AL%, and CA%), the mineralization deposition area was significantly larger (*, *p* < 0.05; **, *p* < 0.01) in group D (the TEBG constructed by BMP-2 overexpressing ASCs and β-TCP) at all the time points, especially at the early time point (2w, as shown in Fig. 6b and c, and 6d). The labeled areas in groups B and C were significantly higher than that of group A at 4w and 8w after implant placement surgery. However, at the earlier phase (2w after implantation operation), the difference among groups A, B, and C has no statistical meaning (*p* < 0.05). There was no difference between groups B and C at all times. Group D had the largest labeled area, as shown by fluorescent trichrome, while group A had the smallest. Groups B and C displayed no significant difference (Fig. 6e, **, *p* < 0.01; ns, no statistic difference).

### Histological and histomorphometric findings

The VG staining showed newly mineralized tissue in all groups, with the largest area in group D and the smallest in group A (Fig. 2a). Also, the new bone morphology was more condensed and mature than that of the other three groups, especially group A. On the other hand, the residual material was easier to detect in group A (Figs. 2a and 3a). The grafted bone substitutes existed in complete shape around the implant surface, and some have also integrated within newly formed bone (as indicated by the yellow arrows in Fig. 2a ⑤). In groups with ASCs, the form of residual material was much smaller. Scattered small black particles were found in groups B and C around the implant surface (Fig. 2a ⑥ and ⑦, yellow arrows), indicating a strengthened remodeling process. Among the four groups, the bone formation process generally coped with their TEBGs degradation process. The histomorphometric analysis showed that the %NB were 6.20 ± 1.08, 18.79 ± 2.38, 23.18 ± 1.26, and 40.48 ± 5.17, respectively (Fig. 2c, **, *p* < 0.01), and the %RM were 11.82 ± 2.6, 5.30 ± 1.76, 6.71 ± 1.72, and 4.65 ± 1.48 respectively. (Fig. 3c) The grafted artificial bone substitute and the newly formed bone structure successively supported the implant’s stability throughout the observation window [20].

**Fig. 7:**
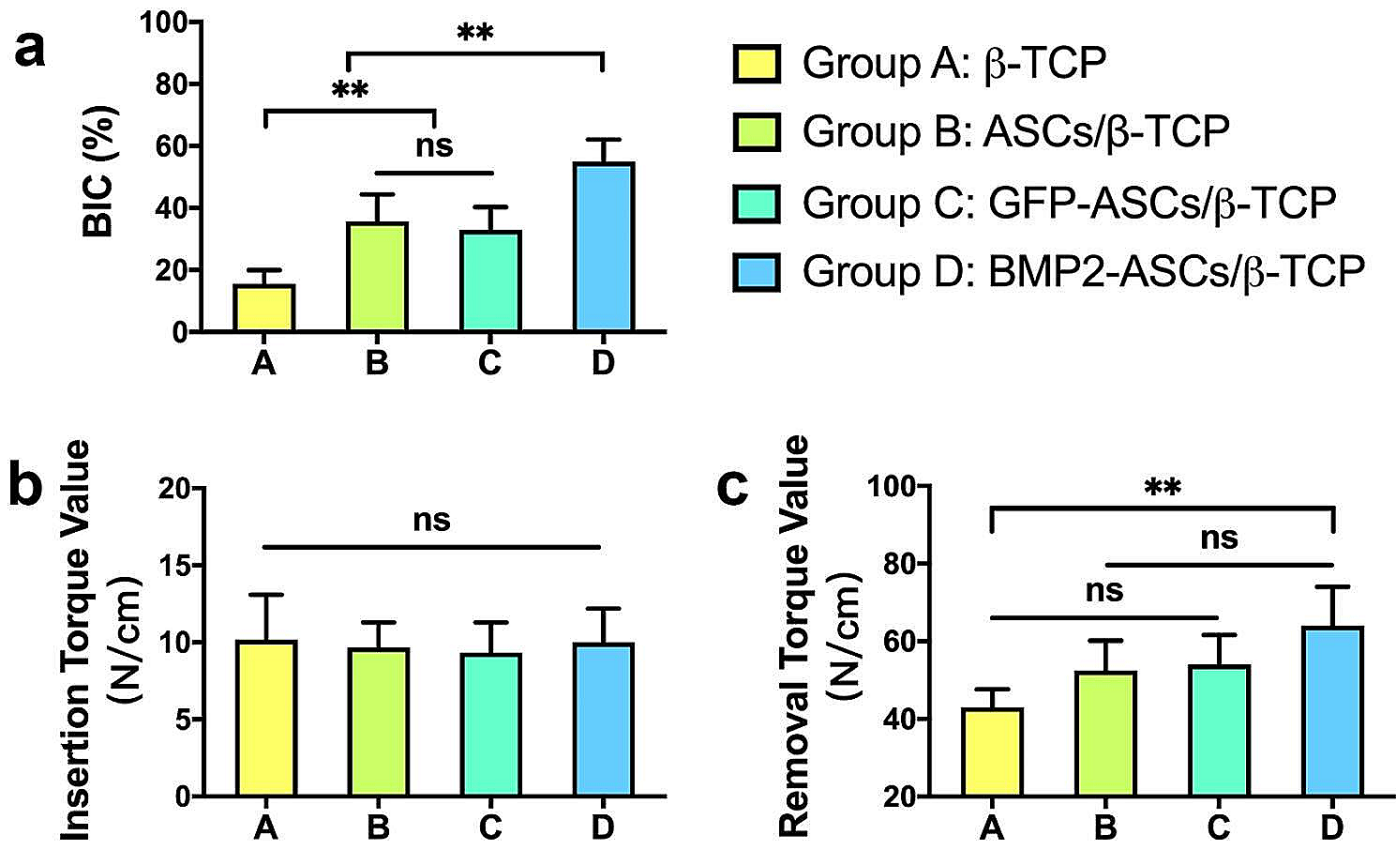
Bone-to-implant contact (BIC) and bone mechanical properties. **a**: %BIC result. **b**: Insertion torque value (ITV, N/cm). **c**: Removal torque value (RTV, N/cm). Recorded ITV of the four groups during the implant placement. After eight weeks of healing, the RTV revealed that mechanical fixation formed in all groups. And the BIC% demonstrated a similar trend as shown by RTV. Group D was significantly more potent than the other groups (**, *p* < 0.01, ns, no significant difference)

Bone-to-implant direct contact was formed in all groups, indicating the osteoconductive and osteoinductive properties of β-TCP and its wedging mechanic function at implant placement. %BIC was 15.5 ± 4.48, 35.71 ± 8.70, 32.94 ± 7.38, and 54.93 ± 7.11 for the four groups, respectively (Fig. 7a). The elevated %BIC in groups B, C, and D further proved the osteogenic capability of TEBGs with ASCs and BMP-2 gene modification.

### Biomechanical analysis

At insertion, all groups demonstrated similar removal torque values, as 10.17 ± 2.93 (N/cm), 9.67 ± 1.63 (N/cm), 9.33 ± 1.97 (N/cm), and 10 ± 2.19 (N/cm), respectively (Fig. 7b, ns, no statistic difference). However, eight weeks later, the integrated implants displayed noticeably higher bonding force between the new bone and the implant. The maximal removal torque force 64 ± 10.04 (N/cm, *p* < 0.05) was detected in group D. The RTV for group A was 43 ± 4.6 (N/cm), while for group B and group C, the mean RTV was 52.5 ± 7.66 (N/cm) and 54 ± 7.64 (N/cm), respectively (Fig. 7c). There was a weak connection between ITV and RTV. The RTV was consistent with previous histological founding, including %TFL, %NB and %BIC.

## Discussion

In this study, we harvested autologous ASCs to construct a BMP-2 overexpressing TEBC [19]. A damaged socket was made on the rabbit femur by making a penetrating osteotomy to minimize the initial sustainability from cortical bone to refer to a peri-implant defect situation [38]. Implantation of low PS was mimicked by inserting an implant into the bone substitute grafted osteotomy. The results substantiated that TEBCs insertion accelerated the calcium deposition on the implant with low PS in the grafted osteotomy. It also proved that ASCs and BMP-2 overexpression promoted earlier osseointegration on low PS implants.

TE has been proven to be one of the most promising strategies in bone regeneration [19, 37, 42]. Classic TE delicately cooperates with three main elements, including scaffolds, growth factors, and seed cells [16]. However, the transplanted cells are not always robust enough to sustain the repairing process, which raises a demand for more vital osteogenic capability from growth factors [43]. Clinically, the most accepted proteins include BMPs [44]. Successful gene therapy allows the entry of recombinant nucleic acid across the plasma membrane, which enables the transfected cells to correct the congenital deficiency or empower more substantial therapeutic capability by up/down-expressing target factors [45, 46]. The combination of TE and gene therapy could easily bypass the up-mentioned difficulties by designing ideal seed cells with the adjustable secretion of endogenous and functional target proteins [22, 47, 48]. Nascent proteins synthesized locally after gene transfer are likely to undergo authentic post-translational modification and have higher activity than recombinant counterparts. Disarmed viral particles have been successfully employed in the past decades for this purpose. Previously, we have utilized the BMP-2 gene- enhanced MSCs through ex vivo transfection by viral vectors for bone regeneration and implant osseointegration [19, 20, 49]. Adipose-derived stem cells (ASCs) has several advantages; isolation high yield, easy access, growth factor secretion, and angiogenic ability and have been widely used in bone regeneration [50, 51]. Our results proved the multi-lineage differentiation properties of ASCs and the enhanced OPN expression of the BMP-2 overexpressing ASCs in vitro, which was consistent with previous studies [19, 20].

Lack of PS is a common situation in clinics, while it does not have a clear definition [14]. Rodrigo, et al. classified the lack of primary stability into four categories [10]. Among them, light rotation with a feeling of resistance, rotation with no resistance, and rotation and lateral oscillation were categorized as having no PS. Other investigators suggested low insertion torque of less than 10 Ncm [3]. In this study, an oversize osteotomy in the femur of rabbits was shaped to mimic a damaged dental implant site in which the implants could rotate with a feeling of resistance after inserting the bone graft. The insertion torque represented the PS as in previous reports [3]. Approximately 250 mg cell–material complex was inserted in each hole to sustain the implant position [37]. This model established an extreme condition of low PS by combining the damaged natural bone, bone grafts and implants to analyze the mechanical changes on the interface of the implant. Some studies used blood clot as the control group to see the natural healing result of the peri-implant defect [38, 39]. However, since we established a damaged socket by making a penetrating osteotomy on the femur to minimize the initial sustainability from cortical bone, it is unpredictable to leave an implant alone in the oversized defect. Meanwhile, the peri-implant defect grafting with autogenous bone or bone graft material in immediate implant placement is regularly applied in clinic [14]. It was also common to apply a widely-used bone substitute as the control group [52, 53]. Thus, the results reflected the changes of histological parameters and bone mechanical properties around implants with low PS in grafted bone substitutes within an oversized osteotomy.

Trichrome fluorescent labeling affords a temporospatial recording of the new bone formation process [54]. Though relatively weak, the fluorescence of the three chromes was observed in group A, respectively. The residual materials were also noticed within the new bone. The result validated the osteoconductive property of the commonly-used bone graft on the low PS. On the other hand, the labeled areas suggested an earlier and stronger mineralization activity of the B, C, and D groups, especially group D. This elevated regeneration has been shown in our previous study on a canine peri-implantitis model [19]. Here, we found that the TE strategy strengthened bone regeneration under a low PS situation on the graft-implant interface, which is particularly important for immediate implantation in the damaged alveolar.

Despite the ever-increasing number of published works on PS and osseointegration, limited evidence is available on the predictability of bone substitute grafted implant sites on the osseointegration process [14, 38]. Since the implant removal torque is one of the relevant factors that impact SS, the present study was undertaken to evaluate the effect of TEBCs variation on the bonding strength at the bone-implant interface [54]. After eight weeks of healing, the RTV revealed that mechanical fixation formed in all groups. And group D was significantly more potent than the other groups. Consistent with our hypothesis, the BIC% demonstrated a similar trend as shown by RTV. The synergic change between BIC% and RTV has also been proved before [55–57].

In the current work, an oversize osteotomy in the femur of a rabbit represented a damaged alveolar socket, which may not completely mimic the complex clinical situations. Also, the effect of constantly changing stress conditions during mastication was ignored. Meanwhile, the established defect only represented a horizon defect without bone height loss, which proves to be a big challenge leading to staged bone regeneration possibilities and a prolonged waiting period. We also have to consider that a direct transfer of the results of this animal study into clinic has to be done with caution [58]. However, this study focused on low PS cases. Our result has shed a light on the implant stability change tuned by the new bone formation of TEBCs on the graft-implant interface.

## Conclusion

In conclusion, new bone formation was accelerated around implants with low primary stability in grafted bone substitutes within an oversized osteotomy. The application of BMP-2 overexpressing ASCs based TEBC promoted earlier osseointegration and more solid bone mechanical properties on low PS implants.

## Data Availability

The data that support the findings of this study are available from the corresponding author upon reasonable request.

## Abbreviations

BIC: Bone-to-implant contact
ITV: Insertion torque value
RTV: Removal torque value
PS: Primary stability
TEBCs: Tissue-engineered bone complexes
TCP-β: Tricalcium phosphateβ
ASCs: Adipose derived-stem cells
TFL: Trichromatic fluorescent labeling
NB: New bone
RM: Residual material
BIC: Bone-implant contact
RTF: Removal torque force
SS: Secondary stability
TS: Total stability
CRA: cutting torque resistance analysis
IT: Insertional torque
RFA: Resonance frequency analysis
MOI: Multiplicity of infection
EGFP: Enhanced green fluorescent protein
TE: Tetracycline
AL: Alizarin red s
CA: Calcein
VG: Van Gieson’s picro-fuchsin
DMEM: Dulbecco’s modified Eagle’s medium:TGF-β3:transforming growth factor-β3
ELISA: enzyme-linked immunosorbent assay
PE: Phycoerythrin
CLSM: Confocal laser scanning microscope
